# Oscillometric central blood pressure and central systolic loading in stroke patients: Short-term reproducibility and effects of posture and fasting state

**DOI:** 10.1371/journal.pone.0206329

**Published:** 2018-11-01

**Authors:** Andrew Mitchelmore, Lee Stoner, Danielle Lambrick, Lucy Sykes, Charlotte Eglinton, Simon Jobson, James Faulkner

**Affiliations:** 1 Department of Sport, Exercise and Health, University of Winchester, Winchester, United Kingdom; 2 Department of Exercise and Sport Science, University of North Carolina at Chapel Hill, Chapel Hill, North Carolina, United States of America; 3 Faculty of Health Sciences, University of Southampton, Southampton, United Kingdom; 4 Hyper-Acute Stroke Unit, Hampshire Hospitals Foundation NHS Trust, Winchester, United Kingdom; University of Perugia, ITALY

## Abstract

**Background:**

This study examined the short-term reproducibility of non-invasive estimates of central and peripheral blood pressure and markers of central systolic loading (augmentation index [AIx; a measure of central systolic loading] and AIx75 [AIx standardised to 75 b·min^-1^ heart rate]) and the effect of posture and fasting state on these variables in patients with acute stroke.

**Methods:**

Twenty-two acute stroke patients (72 ± 10y) had blood pressure measured using the SphygmoCor XCEL in supine and seated postures and whilst fasted and non-fasted.

**Results:**

Acceptable short-term reproducibility (ICC >0.75) was reported for all peripheral and central variables in all conditions (ICC = 0.77–0.90) and for AIx and AIx75 in both fasted postures (ICC = 0.78–0.81). Food consumption significantly lowered all blood pressures (*p* <0.05; η^2^_p_ = 0.20–0.55). The seated posture resulted in a significantly greater AIx than supine (*p* <0.05; η^2^_p_ = 0.22). Fasting state had significant main effects on AIx and AIx75 (*p* <0.05; η^2^_p_ = 0.14–0.22).

**Conslusions:**

Oscillometric estimates of central blood pressure have high short-term reproducibility in different postures and fasting states but markers of systolic load should be assessed whilst fasted. Fasting state has a large effect on central and peripheral blood pressures and on measures of systolic loading. It is important for clinicians to be aware of optimal assessment conditions without this impacting on patient wellbeing.

**Trial registration:**

**Clinical trial registry name**: NCT02537652.

## Introduction

Hypertension is the most common disease seen in primary care [1]. This condition is positively and continuously related to first-time stroke [2–3] due to added haemodynamic stresses on the brain [4]. Controlling hypertension is a cornerstone of recurrent stroke risk reduction [5]. Increased stresses on the brain cause poor neurological recovery after stroke [6] and lead to elevated risk of stroke recurrence [7]. Treating hypertension may be the most important tool in preventing recurrent strokes [8] and maximising quality of life post-stroke.

The assessment of blood pressure is traditionally completed through occlusion of the brachial artery (peripheral blood pressure), but central blood pressure (cBP) measures (either measured directly or derived from peripheral pulse waves) may be more closely related to cardiovascular risk [9]. The invasive measurement of central pressures is usually contraindicated [10], but novel techniques are able to non-invasively estimate central pressures using oscillometric pulse wave analysis (PWA). There is good agreement with PWA and tonometer-based methods of measuring central pressures in patients with atrial fibrillation [11], a frequent indicator of elevated stroke risk. Although oscillometric devices have been demonstrated to be valid [12–15], research is required to report the reproducibility of these devices when assessing central haemodynamic variables before they can be used diagnostically and prognostically in clinical research and treatment settings.

Identifying the optimal operating conditions of devices able to non-invasively calculate central haemodynamic variables in terms of both posture and fasting state is an important step in their introduction to research and clinical use. Posture [16–17] and fasting state [18] have been found to alter peripheral blood pressure measures in non-clinical populations (aged 18-62y). Whilst the acute effects of postural change and fasting state on central blood pressures and central systolic loading (e.g., Augmentation index; AIx) have been investigated in both young and older non-clinical populations [19–21], the short-term reproducibility of these variables has not been investigated in a stroke population. This is important as blood pressures are measured in differing postures and fasting states within clinical settings according to a variety of environmental and situational factors.

This study examined the effect of posture and fasting state on the short-term reproducibility of peripheral and central blood pressures and central systolic loading in an acute stroke population using a non-invasive, oscillometric device (SphygmoCor XCEL). It was hypothesised that posture and fasting state would have a significant effect on peripheral and central blood pressures and markers of central systolic loading and that oscillometric PWA would report high between-day reproducibility in an acute stroke population. These findings will be of importance to those considering the use of the non-invasive oscillometric devices to estimate central blood pressures in research and clinical settings.

## Materials and methods

The methods of this study are reported in accordance with the Helsinki Declaration of 1975 and STROBE (Strengthening the Reporting of Observational Studies in Epidemiology) guidelines [22].

### Participants

**Fig 1:**
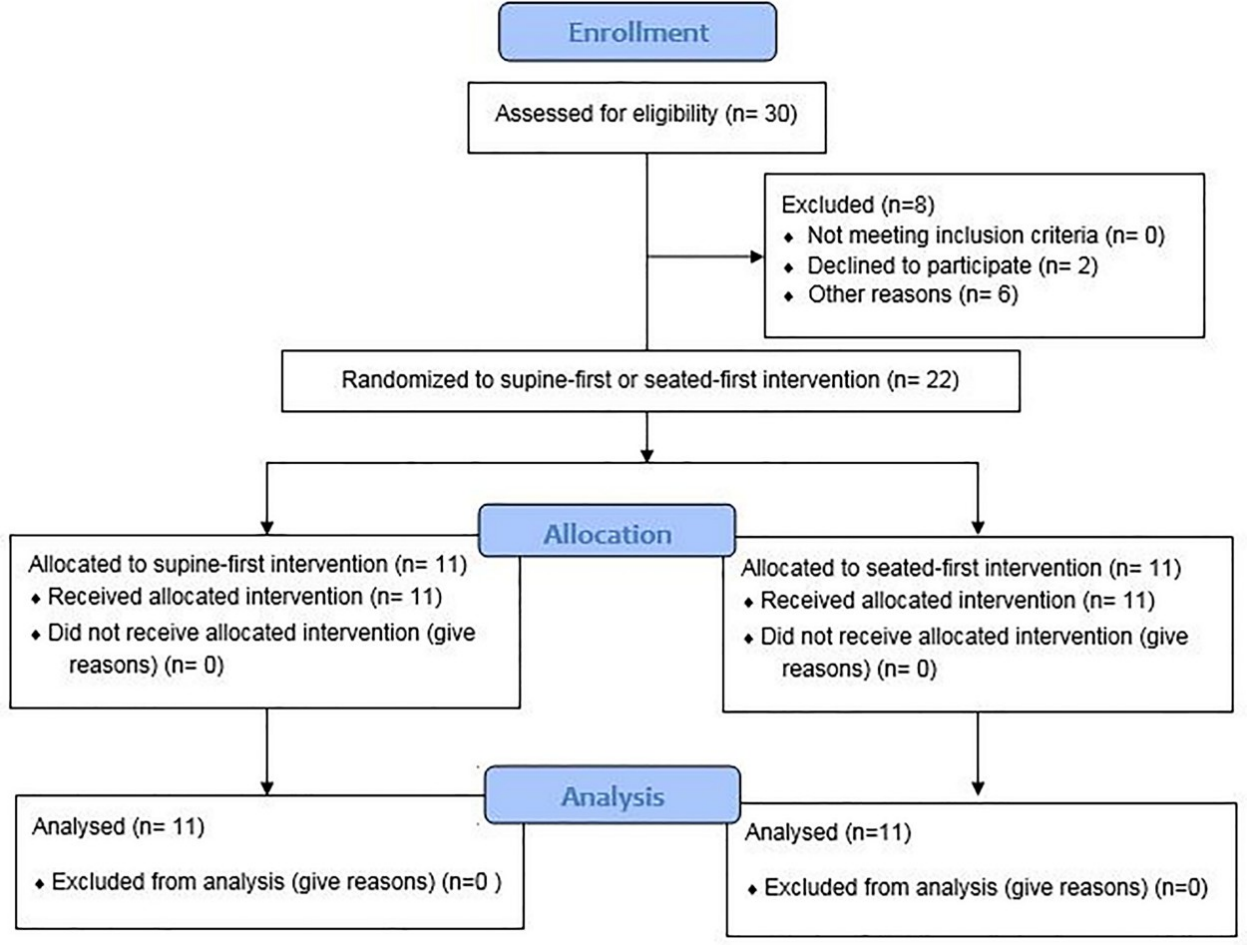
Consort statement.

Twenty-two stroke patients (M = 16; age: 72.3 ± 10.4 y; National Institutes of Health Stroke Scale [NIHSS]: 8.1 ± 5.1; time after stroke: 13.2 ± 12.2 days) provided written consent whilst they were in-patients at a hospital in Hampshire, UK. Recruitment is outlined in Fig 1. Patients were excluded if they were end-of-life stroke patients, had an unstable cardiac condition, were oxygen-dependent, had significant dementia, were unable to swallow normally, lacked capacity to consent, were diagnosed more than eight weeks prior to assessment, had either type I or II diabetes mellitus or were hypoglycaemic at hospital admission. All participants completed a health history questionnaire [23; Table 1]. Ethical approval was granted by the Health Research Authority (REC reference: 15/SC/0559) South Central–Hampshire A Research Ethics Committee. The study was registered as a clinical trial (NCT02537652; https://clinicaltrials.gov/ct2/show/NCT02537652).

**Table 1:** Participant demographic data.

		*n*	%
**Participants**		22	
**Age (y)**		72.3 ±10.4	
**Sex**	Male	16	73
	Female	6	27
**Descent**	European	22	100
**Stroke subtype**	Small vessel lacunar	2	9
	Partial anterior circulation stroke	8	36
	Total anterior circulation stroke	3	14
	Posterior circulation stroke	1	5
	Intracerebral haemorrhage	6	27
	Undetermined	2	9
**Family history of CVD**	Myocardial infarction	9	41
	Heart surgery	1	5
	Stent	0	0
	Catheter	1	5
	Heart defect	1	5
	Stroke	7	32
**Personal history of CVD**	Hypertension	10	45
	High cholesterol	6	27
	Diabetes	0	0
	Coronary artery disease/heart failure	5	23
	Atrial Fibrillation	8	36
**Comorbidities**	Thyroid disease	2	9
	Lung disease	0	0
	Asthma	0	0
	Cancer	2	9
	Kidney disease	1	5
	Hepatitis	2	9
**Lifestyle factors**	Current smoker	2	9
	Previous smoker	6	27
	Current alcohol drinkers	17	77
	Current weight loss plan	1	5
**Everyday activity**	Sedentary	3	14
	Lightly active	3	14
	Moderately active	15	68
	Vigorously active	1	5
**Medication**	Statins	2	9
	Anti-thrombotic	14	64
	Diuretics	1	5
	Calcium blockers	5	23
	Alpha blockers	1	5
	Beta blockers	5	23
	Anticoagulants	1	5
	Other anti-hypertensive medication	5	23
	ACE-I	4	18
	ARB	2	9

Abbrevations: ACE-I–Angiotensin-converting-enzyme inhibitor, ARB–Angiotensin II receptor blockers, CVD–Cardiovascular disease

### Experimental design

Participants were tested on three consecutive mornings, having consumed only water in the 12 hours prior to data collection. After random allocation to a supine-first or seated-first condition using a computerised random number generator, participants assumed this posture in a fasted state for twenty minutes. A minimum of two PWA measurements were completed using the SphygmoCor XCEL (AtCor Medical, Sydney, Australia) with a three minute interval. Measures of PWA consisted of a peripheral blood pressure measure followed by a 10-second sub-systolic recording. The merging points of the forward and reflected waves were identified on the aortic pressure waveform [20]. Augmentation index (AIx) is defined as the augmentation pressure expressed as a percentage of central pulse pressure. If a difference of > 5 mmHg in peripheral blood pressure and a difference of > 4% AIx was recorded (as per manufacturer guidelines), a third measure was completed and an average taken of the closest two. Measurements were taken at heart level in both postures to ensure no changes in AIx were found due to alterations in arm angle. Participants rested for twenty further minutes in the alternative posture before these measures were repeated to complete the fasted condition on each morning. A standard hospital breakfast was consumed (either cereal with milk, a bread roll with marmalade or porridge–all with the option of a small juice) before the protocol was repeated in the same order but in a non-fasted state. Order of fasted state was not randomised due to measurements occurring in a narrow timing window to avoid blood pressure differences caused by circadian rhythms and timing constraints in terms of days of data collection per participant before discharge. This protocol led to the final measures being approximately 45 minutes after food intake. There were approximately eight data points per session, leading to a total of ~528 data points per variable.

### Sample size

A priori sample size calculations were based on central systolic blood pressure measures as the primary outcome and assumed a typical error of 6.4 mmHg adopted from a previous reliability study with healthy participants [24]. The maximum chances of a type 1 or 2 error were set at 5% (very unlikely) and an approximate total of eight participants were required to detect a 6 mmHg change (based on the smallest change reported in previous blood pressure studies [19].

### Statistics

Analyses were run using Statistical Package for Social Sciences v.22 (SPSS, Inc., Chicago, Illinois, USA). All presented data are means (standard deviation, SD). Statistical significance was set at p < 0.05 (two tailed). Analysis of variance for repeated measures with two within-participant factors (posture and fasting state) examined differences in central and peripheral pressures (peripheral systolic blood pressure [SBP], peripheral diastolic blood pressure [DBP], central systolic blood pressure [cSBP], central diastolic blood pressure [cDBP] and central pulse pressure [cPP]) and central systolic loading; AIx standardised to HR of 75 b·min^-1^ [AIx75]). An independent samples t-test was run to ensure that gender had no significant effect on measures of blood pressure, AIx or AIx75 (p > .05). Effect sizes were reported using partial eta squared ($\eta_{p}^{2}$) with 0.01, 0.06 and 0.14 representing small, medium and large effects [25].

The short-term reproducibility of the device was measured by calculating the intra-class correlation coefficient (ICC), standard error of measurement (SEM) and smallest detectable change (SDC; the critical difference in a variable which must be exceeded between two sequential results for a statistically significant change to occur [26]. Excellent reproducibility was reported as an ICC > 0.75 [27].

## Results

Data was successfully collected from all participants in each condition. There were no gender differences in measures of blood pressure, AIx or AIx75 (*p >* .05).

### Central and peripheral blood pressures

When measuring peripheral blood pressure and CBP, the SphygmoCor XCEL reported excellent short-term reproducibility in all variables with ICCs exceeding the 0.75 criterion for excellent reproducibility (ICC = 0.77–0.90; Table 2). No interaction effects were observed. Posture was reported to have a significant main effect on DBP and cDBP (*p =* 0.001; η^2^_p_ = 0.43), with DBP and cDBP both significantly increasing in a seated posture relative to supine. Fasted state had a significant main effect on central and peripheral haemodynamics, with significant decreases in SBP, DBP, pPP, cSBP, cDBP and cPP observed (*p* < 0.05; η^2^_p_ = 0.20–0.55; Table 3 & Supplementary Table).

**Table 2:** Short-term reproducibility of SphygmoCor XCEL in measuring peripheral and central haemodynamic variables.

	Supine-F			Supine-NF			Seated-F			Seated-NF		
	ICC	SEM	SDC	ICC	SEM	SDC	ICC	SEM	SDC	ICC	SEM	SDC
MAP (mmHg)	0.88	4.6	12.7	0.81	5.6	15.4	0.83	5.1	14.3	0.84	5.8	16.0
SBP (mmHg)	0.84	7.3	20.4	0.84	7.8	21.7	0.83	7.3	20.3	0.85	7.6	21.1
DBP (mmHg	0.89	3.6	9.9	0.80	4.5	12.5	0.82	4.5	12.5	0.81	5.3	14.6
PP (mmHg)	0.80	5.4	15.1	0.89	4.9	13.5	0.77	5.8	16.2	0.82	5.7	15.9
cSBP (mmHg)	0.85	6.3	17.4	0.83	7.0	19.4	0.81	6.3	17.5	0.83	7.0	19.4
cDBP (mmHg)	0.90	3.5	9.7	0.82	4.4	12.2	0.83	4.4	12.2	0.83	5.1	14.2
cPP (mmHg)	0.84	4.1	11.4	0.88	3.8	10.4	0.79	4.5	12.6	0.83	4.3	11.9
Heart rate (b·min^-1^)	0.89	3.3	9.2	0.83	4.4	12.3	0.88	3.8	10.6	0.85	4.0	11.0
AP (mmHg)	0.76	2.8	7.8	0.66	3.0	8.4	0.72	3.5	9.7	0.71	2.9	8.1
AIx (%)	0.81	3.7	10.3	0.66	5.1	14.0	0.78	5.1	14.2	0.73	5.1	14.1
AIx75 (%)	0.81	4.7	12.9	0.70	5.6	15.4	0.78	6.2	17.1	0.74	5.8	16.1

Abbrevations: Abbreviations: AIx—Augmentation Index, AIx75—Augmentation Index @ 75bpm, AP = Augmented Pressure, cDBP—Central Diastolic Blood Pressure, cPP—Central Pulse Pressure, cSBP—Central Systolic Blood Pressure, DBP—Diastolic Blood Pressure, F–Fasted, ICC–Intraclass Correlation Coefficient, MAP—Mean Arterial Pressure, NF—Non-Fasted, SDC–Smallest Detectable Change, SEM–Standard Error of Measurement, SBP—Systolic Blood Pressure

**Table 3:** Mean and SD for peripheral and central haemodynamic variables.

		Total	Supine		Seated		Interaction		Posture		Fasted	
			Fast	Non	Fast	Non	*P*	η^2^_p_	*P*	η^2^_p_	*P*	η^2^_p_
MAP (mmHg)	$\bar{X}$	100	104	94	106	97	.86	.00	**.003**	.34	**< .001**	.52
	*SD*	*12*	*13*	*13*	*12*	*15*	* *	* *	* *	* *	* *	* *
SBP (mmHg)	$\bar{X}$	142	147	137	148	138	.82	.00	.09	.14	**< .001**	.48
	*SD*	*18*	*18*	*20*	*18*	*20*	* *	* *	* *	* *	* *	* *
DBP (mmHg)	$\bar{X}$	79	82	74	85	76	.90	.00	**.001**	.43	**< .001**	.53
	*SD*	*10*	*11*	*10*	*10*	*12*	* *	* *	* *	* *	* *	* *
PP (mmHg)	$\bar{X}$	64	65	63	64	62	.57	.02	.33	.05	**.04**	.20
	*SD*	*13*	*12*	*14*	*12*	*13*	* *	* *	* *	* *	* *	* *
cSBP (mmHg)	$\bar{X}$	130	135	123	137	124	.82	.03	.06	.16	**< .001**	.59
	*SD*	*15*	*16*	*17*	*15*	*17*	* *	* *	*** ***	* *	* *	* *
cDBP (mmHg)	$\bar{X}$	80	83	75	86	78	.95	.00	**.001**	.43	**< .001**	.50
	*SD*	*10*	*11*	*10*	*11*	*12*	* *	* *	* *	* *	* *	* *
cPP (mmHg)	$\bar{X}$	49	52	48	51	47	.71	.01	.06	.16	**< .001**	.55
	*SD*	*10*	*10*	*11*	*10*	*10*	* *	* *	* *	* *	* *	* *
Heart rate (b·min^-1^)	$\bar{X}$	68	65	68	66	71	.30	.05	**.04**	.19	**< .001**	.60
	*SD*	*10*	*10*	*11*	*11*	*10*	* *	* *	* *	* *	* *	* *
AP (mmHg)	$\bar{X}$	16.2	18.8	14.9	17.5	13.7	.65	.01	**.02**	.22	**< .001**	.61
	*SD*	*5*.*4*	*5*.*8*	*5*.*2*	*6*.*6*	*5*.*5*	* *	* *	* *	* *	* *	* *
AIx (%)	$\bar{X}$	31.9	35.5	30.3	33.5	28.4	.97	.00	**.02**	.22	**.001**	.43
	*SD*	*8*.*7*	*8*.*6*	*8*.*6*	*10*.*9*	*9*.*7*	* *	* *	* *	* *	* *	* *
AIx75 (%)	$\bar{X}$	28.2	30.7	27.2	28.7	26.4	.54	.02	.08	.14	**.03**	.20
	*SD*	*10*.*8*	*10*.*8*	*10*.*1*	*13*.*0*	*11*.*4*	* *	* *	* *	* *	* *	* *

Abbrevations: Abbreviations: AIx—Augmentation Index, AIx75—Augmentation Index @ 75bpm, AP = Augmented Pressure, cDBP—Central Diastolic Blood Pressure, cPP—Central Pulse Pressure, cSBP—Central Systolic Blood Pressure, DBP—Diastolic Blood Pressure, F—Fasted, N—Non-Fasted, MAP—Mean Arterial Pressure, PP–Pulse Pressure, SBP—Systolic Blood Pressure. **Bolded** *P* < .05

### Central systolic loading

When assessing AIx and AIx75, the SphygmoCor XCEL device reported excellent short-term reproducibility in both fasted postures (ICC = 0.78–0.81) and moderate reproducibility in both non-fasted postures (ICC = 0.66–0.74 [Table 2]). Posture had a significant main effect on AIx, with a significant decrease observed in the seated posture (*p* = 0.024; η^2^_p_ = 0.22) but not in AIx75; suggesting that these differences were mainly due to the significant changes in heart rate observed in this study. Fasting state had a significant main effect on both AIx and AIx75 with significant decreases reported after food consumption (*p* < 0.05; η^2^_p_ = 0.14–0.22 [Table 3 & Supplementary Table]).

## Discussion

The SphygmoCor XCEL exhibits high short-term reproducibility in different fasting states and postures when assessing peripheral and central blood pressure measures, but central systolic loads were more reproducible in a fasted than non-fasted state. Fasting state was demonstrated to have a large influence on both peripheral and central blood pressure and central systolic load measures, whereas posture significantly influenced peripheral and central diastolic measures and AIx, but no other variables recorded. The lack of statistical differences in AIx75 between postures suggests that differences in AIx are caused by fluctuations in heart rate caused by postural change. This is in line with previous research showing that AIx is confounded by the timing of the reflected wave [28]. When measuring peripheral and central blood pressures in a stroke population, patients should be in a fasted state to optimise the accuracy and reproducibility of collected data. If patients are non-fasted, it is important that researchers and clinicians are aware of the immediate effects of food intake on these measures and analyse these blood pressure measures accordingly. Due to the high reproducibility and the demonstrated effect of posture and fasting state on central haemodynamic variables, the experimental hypotheses of this study were accepted.

### Central and peripheral blood pressure

High short-term reproducibility was reported when assessing central and peripheral blood pressure measures, with ICCs exceeding the 0.75 criterion of excellence in all conditions. These ICCs (0.77–0.90) are consistent with, but slightly better than, previous work examining the short-term reproducibility of the SphygmoCor XCEL in a younger, healthy population which reported ICCs of 0.68–0.90 for peripheral and central measures [20]. To our knowledge, no other work has been completed determining the short-term reproducibility of this device in an acute stroke population. Based upon the ICC analysis, this study suggests that non-invasive measures may be suitable for the assessment of central haemodynamics. However, it is interesting to note that the SDCs were wider than those reported using the same device in a young, healthy population [20].

No significant interaction effects were reported. Significant main effects were observed for both posture and fasting state on peripheral and central blood pressures. Due to technological advances, the measurement of these variables non-invasively may become widespread. As a result, research into factors influencing central measures is of great importance. It is possible that medications may induce different responses between peripheral and central blood pressure measures [29]. The significant increase in DBP and cDBP in a seated position compared to supine mirrors the findings of previous work [16, 30]. This, alongside a non-significant change in systolic measures, caused a non-significant decrease in pulse pressure. As cPP is recognised as being extremely relevant to vascular ageing [31], the significant influence of posture on cDBP is particularly relevant.

Fasting state was demonstrated to cause statistical decreases in SBP, DBP, pPP, cSBP, cDBP and cPP. A post-prandial decrease of ~10 mmHg was reported in SBP and ~12 mmHg in cSBP. A smaller decrease in DBP (8–9 mmHg) and cDBP (8 mmHg) caused a large change in pPP and cPP to occur. Significant decreases in central blood pressure after food consumption have been observed in a non-clinical population over the age of 50 [21] but not in a young, healthy sample [20]. This suggests that healthy populations may be able to make necessary autonomic adjustments to redirect blood flow without a drop in central blood pressure, but older and clinical populations may be less able to do so effectively. A post-prandial drop in cBP variables was observed by Ahuja *et al* [19] who reported a decrease of 6.1 mmHg after food and water consumption compared to water alone but recruited a wide-ranging sample aged 21–80 years old. After food consumption, this drop in blood pressure may be due to a post-prandial reduction in arterial stiffness in the splanchnic bed, allowing cardiac output to be maintained alongside a decrease in blood pressure. Ahuja and colleagues [19] suggest a peak time-frame of 45 minutes for a blood pressure drop after food intake; indicating that the data reported in this study may reflect the greatest changes in a post-prandial state. It is worth noting that as well as physiological adaptations, these changes in blood pressure may be contributed to by the presence of regression to the mean effect due to the repeated measures taken; a potential bias which is inevitably present for as long as there is less-than-perfect repeatability in the measurement of BP [32]. It should be at the discretion of consultants as to how these optimal operating conditions are balanced against practical patient care, with nutritional strategies adopted to avoid poor outcomes and prolonged stays in hospital [33].

### Central systolic loading

This study reports that the SphygmoCor XCEL has high short-term reproducibility when reporting AIx and AIx75, particularly in fasted participants. ICCs of 0.78–0.81 were observed when recording AIx and AIx75 in a fasted state, whereas this lowered to 0.66–0.73 and 0.70–0.74 for AIx and AIx75, respectively, when participants were non-fasted. The digestive process causes alterations in vasodilation which may vary on a day-to-day or meal-to-meal basis depending on extraneous factors (e.g., meal composition, temperature, hydration status). This may cause the assessment of AIx and AIx75 to become less stable when the body is not truly at rest as it would be more likely to be in a fasted state. The significant main effect observed for posture in AIx but not in AIx75 may add credence to the concept that AIx and HR may not be entirely linearly related; a suggestion which reduces the propriety of AIx75 being reported without AIx alongside [34]. Significant changes to AIx and AIx75 in different postures have not been observed in previous work in a healthy, young population [20]. However, a significant change in AIx but not AIx75 has also been reported in hypertensive participants over the age of 50 [21] but not in the normotensive sample in the same study, who demonstrated significant differences in both AIx and AIx75 in supine and seated postures. A reduction in AIx75 has been reported in a supine state compared to a seated position in a female-only population [35]. Such a finding was not mirrored in this study, with measurements of AIx and AIx75 being ¬1–2% lower in the seated posture compared to supine. This finding was not statistically significant (*p* = 0.08), whilst wide ranges in the reported 95% confidence intervals were reported (see Supplementary Table). This may be due to a potential lack of statistical power to detect an association of this magnitude in this sample of 22 stroke patients. The finding that fasting state had a significant main effect on AIx and AIx75 with post-prandial reductions observed in both measures aligns with previous research and may be a result of increased arterial compliance due to tone alterations in the small vessel beds, large artery function and large artery geometry [20].

### Clinical significance

This study suggests that non-invasive central blood pressure assessments provide reproducible measurements of peripheral and central haemodynamics. Significant decreases in peripheral and central blood pressures were observed after food consumption. During hospitalisation after stroke, assessments of central and peripheral blood pressures should therefore be assessed in a fasted state to reduce the variability caused by food intake. This is particularly true when medication prescription is at least partially based on these routine blood pressure measures. The combined effect of post-stroke medication and fasting state should also be considered when monitoring patient health, as both variables cause a decrease in peripheral and central blood pressure measures.

With regards to central systolic loading, increased arterial stiffness is reported to be significantly associated with reduced cognitive function in stroke patients [36]. Reporting this decrease in central systolic loading in terms of prandial state may have some importance with regards to perfusion pressures and the timing and assessment of cognitive state examinations in a clinical setting around meal times. Further work should investigate any potential links between arterial stiffness, perfusion pressures and cognitive performance both before and after food intake in clinical populations.

### Limitations and strengths

It is important to contextualise the study through the recognition of strengths and weaknesses. Firstly, we did not recruit a unisex sample, a fact which may influence results due to potential differences in responses to postural changes in peripheral blood pressure between sexes [37]. Secondly, due to stringent exclusion criteria and subsequent slow levels of recruitment, we recruited a sample with a range of stroke subtypes and severity according to NIHSS (range: 1–18). Further work should examine whether there are differences in the reproducibility of the SphygmoCor XCEL in more severe strokes, and between stroke subtypes. The study was also not able to take into account the effect of body mass or body composition variables on changes of peripheral and central haemodynamics as patients were not routinely weighed on admission. Finally, the sample contained participants with and without atrial fibrillation; a condition which may lead to some inconsistencies in measured data due to inconsistent stroke volumes. However, biases in oscillometric assessment of central blood pressure have been shown to not significantly differ in the presence or absence of AF when three repeated measures are taken [38], as happened in at least some conditions for every participant in this study. Focusing on central blood pressure assessments in those specifically with atrial fibrillation has the potential to be an interesting area of future study. However, the time-frames involved in the data collection process ensured that post-prandial measures after food consumption were in accordance with recommendations set out by Ahuja and colleagues [19] in terms of capturing the peak effects of food intake on haemodynamic variables. Additionally, the in-patient nature of the study ensured a controlled environment for data collection to occur. Furthermore, the randomisation of condition order and standardised overnight fast also contributed to a strong protocol. Finally, assessments occurred at the same time each day, reducing the likelihood of circadian rhythms altering the blood pressures recorded in the morning.

In conclusion, this study demonstrates that the SphygmoCor XCEL, a non-invasive oscillometric PWA device, possesses high short-term reproducibility in assessing both central and peripheral blood pressure measures in both fasted and non-fasted states, and good short-term reproducibility when assessing markers of central systolic loading, particularly in a fasted state. The current study demonstrates that posture has a significant effect on DBP, cDBP and AIx, whereas, fasting state significantly influences all peripheral and central variables, as well as both AIx and AIx75, in acute stroke patients. The findings of this study are pertinent to researchers and clinicians, although consideration around the practicalities of implementing these measures within practice (e.g. optimising conditions for BP assessment whilst minimising adverse events associated with fasting state) is necessary.

## Supporting information

S1 CONSORT Checklist Mitchelmore consort checklist. (DOC)

S1 Dataset S1_Raw_Data_File. (XLSX)

S1 Table (DOCX)

S1 File Mitchelmore protocol PLOS_ONE. (DOCX)
