# Correction: Oscillometric central blood pressure and central systolic loading in stroke patients: Short-term reproducibility and effects of posture and fasting state

**DOI:** 10.1371/journal.pone.0208477

**Published:** 2018-11-29

**Authors:** Andrew Mitchelmore, Lee Stoner, Danielle Lambrick, Lucy Sykes, Charlotte Eglinton, Simon Jobson, James Faulkner

There are errors in Table 3. The mean and standard deviation values in the third, fourth, fifth, sixth, and seventh columns are incorrect. Please see the corrected Table 3 here.

**Table 3:** Mean and SD for peripheral and central haemodynamic variables.

		Total	Supine		Seated		Interaction		Posture		Fasted	
			Fast	Non	Fast	Non	*P*	η^2^_p_	*P*	η^2^_p_	*P*	η^2^_p_
MAP (mmHg)	$\bar{X}$	99	102	93	105	96	.86	.00	**.003**	.34	**< .001**	.52
	*SD*	*14*	*14*	*13*	*13*	*15*	* *	* *	* *	* *	* *	* *
SBP (mmHg)	$\bar{X}$	142	146	136	149	138	.82	.00	.09	.14	**< .001**	.48
	*SD*	*19*	*19*	*20*	*17*	*19*	* *	* *	* *	* *	* *	* *
DBP (mmHg)	$\bar{X}$	78	81	73	84	76	.90	.00	**.001**	.43	**< .001**	.53
	*SD*	*12*	*11*	*10*	*11*	*12*	* *	* *	* *	* *	* *	* *
PP (mmHg)	$\bar{X}$	66	67	65	67	64	.57	.02	.33	.05	**.04**	.20
	*SD*	*16*	*16*	*17*	*16*	*17*	* *	* *	* *	* *	* *	* *
cSBP (mmHg)	$\bar{X}$	129	134	122	136	124	.82	.03	.06	.16	**< .001**	.59
	*SD*	*17*	*16*	*17*	*14*	*17*	* *	* *	*** ***	* *	* *	* *
cDBP (mmHg)	$\bar{X}$	80	82	74	85	77	.95	.00	**.001**	.43	**< .001**	.50
	*SD*	*12*	*11*	*11*	*11*	*12*	* *	* *	* *	* *	* *	* *
cPP (mmHg)	$\bar{X}$	50	52	48	51	47	.71	.01	.06	.16	**< .001**	.55
	*SD*	*10*	*10*	*11*	*10*	*10*	* *	* *	* *	* *	* *	* *
Heart rate (b·min^-1^)	$\bar{X}$	67	65	68	66	70	.30	.05	**.04**	.19	**< .001**	.60
	*SD*	*11*	*10*	*11*	*11*	*10*	* *	* *	* *	* *	* *	* *
AP (mmHg)	$\bar{X}$	16.6	19.5	15.2	17.7	14.0	.65	.01	**.02**	.22	**< .001**	.61
	*SD*	*6*.*4*	*6*.*6*	*5*.*6*	*6*.*8*	*5*.*6*	* *	* *	* *	* *	* *	* *
AIx (%)	$\bar{X}$	32.3	35.9	30.9	33.7	28.8	.97	.00	**.02**	.22	**.001**	.43
	*SD*	*10*	*8*.*7*	*9*.*6*	*10*.*8*	*9*.*9*	* *	* *	* *	* *	* *	* *
AIx75 (%)	$\bar{X}$	28.6	30.9	27.8	29	26.6	.54	.02	.08	.14	**.03**	.20
	*SD*	*11*.*3*	*10*.*8*	*10*.*6*	*13*.*0*	*11*.*2*	* *	* *	* *	* *	* *	* *

Abbrevations: Abbreviations: AIx—Augmentation Index, AIx75—Augmentation Index @ 75bpm, AP = Augmented Pressure, cDBP—Central Diastolic Blood Pressure, cPP—Central Pulse Pressure, cSBP—Central Systolic Blood Pressure, DBP—Diastolic Blood Pressure, F—Fasted, N—Non-Fasted, MAP—Mean Arterial Pressure, PP–Pulse Pressure, SBP—Systolic Blood Pressure. **Bolded** *P* < .05
