# Supplementary material for: Oscillometric central blood pressure and central systolic loading in stroke patients: Short-term reproducibility and effects of posture and fasting state
Source: PLoS One. 2018 Nov 1;13(11):e0206329. doi: 10.1371/journal.pone.0206329 (PMC6211701; doi:10.1371/journal.pone.0206329)
Supplement: S1 Table — (DOCX) [file pone.0206329.s003.docx]

**Supplementary Table: Mean (**$\bar{\boldsymbol{X}}$**) values and 95% confidence intervals (C.I) from outcome variables for posture and fasting state**

|  |  |  |  |  | |  |  | |  |  |
| --- | --- | --- | --- | --- | --- | --- | --- | --- | --- | --- |
|  |  |  | Posture |  |  |  |  | Fasting state |  |  |
| Variable | Supine $\bar{X}$ | Seated $\bar{X}$ | $\bar{X}$ difference | *P* value for posture | Posture 95% C.I | Fasted $\bar{X}$ | Non-fasted $\bar{X}$ | $\bar{X}$ difference | *P* value for fasting state | Fasting state 95% C.I |
| SBP (mmHg) | 141.4 | 143.2 | 1.8 | .09 | -4.0 - .29 | 147.5 | 137.1 | 10.4 | **< .001** | 5.5 – 15.3 |
| DBP (mmHg) | 77.0 | 79.8 | 2.8 | **.001** | -4.2 - -1.3 | 82.4 | 74.4 | 8.0 | **< .001** | 4.6 – 11.6 |
| PP (mmHg) | 66.3 | 65.4 | 0.9 | .33 | - 1.0 – 2.8 | 67.0 | 64.6 | 2.4 | **.04** | .2 – 4.7 |
| cSBP (mmHg) | 128.2 | 130.0 | 1.8 | .06 | -3.7 - .09 | 135.0 | 123.1 | 11.9 | **< .001** | 7.4 – 16.3 |
| cDBP (mmHg) | 78.0 | 81.0 | 3.0 | **.001** | -4.5 - -1.4 | 83.5 | 75.6 | 7.9 | **< .001** | 4.3 – 11.5 |
| cPP (mmHg) | 50.2 | 49.1 | 1.1 | .06 | -.1 – 2.4 | 51.8 | 47.6 | 4.2 | **< .001** | 2.5 – 5.9 |
| AIx (%) | 33.4 | 31.3 | 2.1 | **.02** | .3 – 4.0 | 34.8 | 29.9 | 4.9 | **.001** | 2.4 – 7.5 |
| AIx75 (%) | 29.4 | 27.8 | 1.6 | .08 | -.2 – 3.4 | 30.0 | 27.2 | 2.8 | **.03** | .30 – 5.2 |

1. Abbrevations: Abbreviations: AIx - Augmentation Index, AIx75 - Augmentation Index @ 75bpm, cDBP - Central Diastolic Blood Pressure, C.I – Confidence Interval cPP - Central Pulse Pressure, cSBP - Central Systolic Blood Pressure, DBP - Diastolic Blood Pressure, PP – Pulse Pressure, SBP - Systolic Blood Pressure
2. **Bolded** *P*<.05
