# Supplementary material for: Oscillometric central blood pressure and central systolic loading in stroke patients: Short-term reproducibility and effects of posture and fasting state
Source: PLoS One. 2018 Nov 1;13(11):e0206329. doi: 10.1371/journal.pone.0206329 (PMC6211701; doi:10.1371/journal.pone.0206329)
Supplement: S1 File — (DOCX) [file pone.0206329.s004.docx]

**Study Title:** **Oscillometric central blood pressure and central systolic loading in stroke patients: Short-term reproducibility and effects of posture and fasting state**

Short sentence: Reproducibility in central blood pressure evaluation in stroke patients

**REC reference: 15/SC/0559**

| Chief Investigator: | Mr Andrew Mitchelmore, University of Winchester, Department of Sport, Exercise and Health, Andrew.mitchelmore@winchester.ac.uk |
| --- | --- |
| Investigators: | Dr Lee Stoner, University of North Carolina at Chapel Hill, Department of Exercise and Sport Science, Stonerl@email.unc.edu  Dr Danielle Lambrick, University of Southampton, Faculty of Health Sciences, D.m.lambrick@soton.ac.uk  Dr Lucy Sykes, Hampshire Hospitals Foundation NHS Trust  Charlotte Eglinton, Hampshire Hospitals Foundation NHS Trust  Professor Simon Jobson, University of Winchester, Department of Sport, Exercise and Health, Simon.Jobson@winchester.ac.uk  Dr James Faulkner, University of Winchester, Department of Sport, Exercise and Health, James.Faulkner@winchester.ac.uk |
| Sponsor: | University of Winchester |

**BACKGROUND AND RATIONALE**

Stroke is a leading cause of death worldwide, and is a prominent cause of chronic disability, causing significant physical, cognitive and social impairment (CDC, 2012; Lozano et al., 2012; Go et al., 2013; Faulkner et al., 2014). In the United Kingdom, there are 150,000 strokes each year, with ~900,000 people living with the debilitating effects of stroke (Go et al., 2013; Stroke Association, 2015). Individuals who experience a stroke or transient ischaemic attack (TIA; minor stroke) are at heightened risk of experiencing vascular events in the future, such as myocardial infarction, stroke and secondary TIAs, and death (Albers et al., 2002; Touze et al., 2005; Giles & Rothwell, 2007).

Of all the measures of interest, greater blood pressure control is the most important contributor to positive health outcomes in stroke patients (Adams et al., 2003). However, clinical practice, and research to date (Faulkner et al., 2013, 2014; Woolley et al., 2014) concentrate on the assessment of peripheral blood pressure (the blood pressure as identified from the arm). The measurement of central haemodynamic parameters, including central systolic blood pressure (cSBP) and arterial wave reflection (i.e., augmentation index, AIx), hold the potential to provide clinicians with important diagnostic and prognostic information beyond that provided by traditional peripheral blood pressure readings. The assessment of central blood pressure is important as it reflects the stress and loading on the left ventricle and coronary arteries (Roman et al., 2007; Young et al., 2015). Considering the marked differences in pulse pressure between the central aorta and peripheral limbs, peripheral blood pressure may not accurately reflect the effects of peak arterial blood pressure on centrally located organs (Protogerou et al., 2007). Previous research has demonstrated central blood pressures to be 50 % superior to peripheral blood pressures when predicting cardiovascular events (Roman et al., 2007), while in comparison to peripheral blood pressure, central blood pressure may improve the management of hypertension (Sharman et al., 2013). Owing to recent technological advances, central haemodynamic parameters can be obtained quickly and noninvasively using an automated blood pressure cuff (oscillometer).While the validity of oscillometric devices which measure central haemodynamic parameters has been demonstrated, further study is required to determine measurement precision (reliability) under normal clinical operating conditions (i.e. in the hospital or GP practice setting).

The purpose to this study is to therefore assess the influence of fasting status (fasted vs. non-fasted) and posture (seated vs. supine) on central and peripheral blood pressure measures in patients diagnosed with stroke. The study will determine whether these parameters can be reliably assessed in an acute stroke population.

**STUDY DESIGN**

**Design and Setting:**

This study is a single centre observational study. Twenty-five stroke patients will be recruited from the Royal Hampshire County Hospital within the Hampshire Hospital NHS Foundation Trust (HHFT).

Patients will be recruited from the inpatient setting. Recruitment and all assessments will take place on the hyper-acute stroke ward in the hospital. Written informed consent will be obtained from all participants.

All stroke patients will be assessed using the National Institute of Health Stroke Severity Scale (NIHSS) by a qualified stroke consultant. Participants will comply with pharmacological treatment as recommended.

Participants will take part in three separate assessment sessions, on three separate days, with a 24 hour recovery between each session. Each assessment is expected to last 90 minutes, with a minimum of eight blood pressures being taken from the upper arm. As such, participants will be asked to give up 4.5 hours of their time to the study. During each assessment participants will be tested in a fasted and non-fasted state, and in a supine (lying) and seated position. All assessments will take place between 7 and 10am and will be undertaken following written informed consent. On-going assessment of capacity to consent will take place throughout the data collection process on each day.

**Primary and Secondary Endpoints/Outcome Measures**

The primary outcome measure is central systolic blood pressure.

Secondary outcome measure will include all other blood pressure parameters recorded (peripheral systolic blood pressure, pulse pressure, augmentation index and double product), from the blood pressure device (for further information see paragraph below).

Oscillometric pressure waveforms will be recorded on the upper arm using the SphygmoCor XCEL device (AtCor Medical, Sydney, Australia) during all assessments, following standard manufacturer guidelines. Each measurement cycle lasts approximately 60 sec, consisting of a brachial blood pressure recording and then a 10 sec sub-systolic recording. A corresponding aortic pressure waveform will then be generated using a validated transfer function, from which central systolic, diastolic, pulse pressure (cSBP, cDBP, cPP), augmentation pressure (AP), and augmentation index (AIx) will be derived. The AP is defined as cSBP minus the pressure at the inflection point, whereby the inflection point is the merging of the forward and reflected waves. The AIx is defined as the AP expressed as a percentage of cPP. AIx is influenced by heart rate, and thus an index corrected for a heart rate at 75 beats per minute (AIx@75) will also be calculated.

Central and peripheral blood pressures will be assessed in the following four conditions during each of the three assessment sessions: i) fasted, supine, ii) fasted, seated, iii) non-fasted supine, and iv) non-fasted seated. During each of the above conditions two measurements will be taken, separated by a three-minute interval. If blood pressure differ by > 5mmHG a third recording will be taken and the closest recordings will be averaged. Each of the above conditions will be undertaken following a minimum 15 minutes quiet rest.

**Study** **Participants**

Participants must meet the study’s inclusion criteria, and must not meet any of the study exclusion criteria to be able to participate in the study. All participants will have been diagnosed with stroke by a stroke consultant at the Royal Hampshire County Hospital.

Inclusion Criteria

1. Patients diagnosed with stroke
2. Reside within the locality of the Hampshire Hospital's Foundation Trust
3. Patients are to be assessed within 8 weeks of their initial stroke diagnosis.
4. Do not meet exclusion criteria

Exclusion Criteria

The participant may not enter the study if ANY of the following apply:

1. End-of-life stroke patients
2. Unstable cardiac conditions
3. Oxygen dependence
4. Significant dementia
5. Unable to swallow
6. Patients lacking capacity to consent to participate in the study (capacity will continue to be assessed throughout data collection)
7. Patient is diagnosed with stroke more than 8 weeks prior to an assessment.
8. Type I or II diabetic stroke patients
9. Hypoglycaemic at hospital admission

**Study Procedures**

Participants will take part in three separate assessment sessions, on three separate days, with a minimum 24 hour recovery between each session. Before each assessment participants will undertake an overnight fast (only water is allowed to be consumed prior to the assessment). Participants will take their medication the morning of each assessment in accordance with the normal pharmacological management procedures which are used on the acute stroke ward at the Royal Hampshire County Hospital. Following this, participants will rest in either a supine or seated posture for a minimum of 15 minutes. Oscillometric pressure waveforms (using the SphygmoCor XCEL), recorded on the left upper arm, will assess haemodynamic parameters including central and peripheral blood pressure, pulse pressure, and augmentation index. Two measurements will be taken with a three-minute interval. If differences in peripheral blood pressure are > 5 mmHg or augmentation index are > 4 %, a third measure will be taken. Following this, participants will be moved to the alternate body position and remain rested for a further 15 minutes. Identical procedures to those outlined above will then be used to assess central and peripheral blood pressure responses. Thereafter, patients will consume their normal breakfast. However, caffeine cannot be consumed during this time. Thirty-minutes after food consumption participants will undertake identical procedures as those outlined above in both a non-fasted-supine and non-fasted-seated conditions.

Identical procedures to those outlined in the first assessment session will be used to assess central and peripheral blood pressure measures during the second and third assessment day.

**Participant Confidentiality**

All personal data collected will be entered centrally on to a computer in alphanumeric code. The study staff will ensure that the participants’ anonymity is maintained. The participants will be identified only by initials and a participants ID number on the electronic database. Data will be securely stored on a password protected network, which can only be accessed by the researchers involved in the study. Raw data will be locked in a filing cabinet immediately after any patient assessment, located in Prof. Jobson and Dr Faulkner’s office (Centre of Sport, Room 105) on the University of Winchester's King Alfred’s Campus. This data will not be taken out of the office.

**Other Ethical Considerations**

Patients who have special communication needs will be supported during the study (i.e., patients with dysphasia, deaf). The stroke team and research team (from the University of Winchester) will have completed appropriate training regarding how to discuss the study with patients with dysphasia. The research team have met with Speech and Language Therapists from HHFT (Suzanne Ramos) to discuss issues surrounding consenting patients with dysphasia, and are due to attend an aphasia course on 9th November 2015 (UK connect).

Whilst the participant information will be in English, the HHFT's translation and interpreting services will be used (as is usual practice in clinical care settings) when English is not a patient's first language.

**Data Handling and Record Keeping**

All study data will be entered on a registered Microsoft Office computer at the University of Winchester. Data will be analysed as group means and individual participants identities will not be disclosed on any documentation (other than the signed consent form). The participants will be identified by a study specific participants number and/or code in any database. The name and any other identifying detail will NOT be included in any study data electronic file. The data will be kept for 7 years by Dr Jobson and Dr Faulkner to allow investigators to return to the source of the data if/when needed. As academic supervisors to Mr Andrew Mitchelmore (PhD researcher), Dr Jobson and Dr Faulkner will be responsible for ensuring that all data is kept securely and disposed of appropriately following this timeframe. All physical documents will be shredded after the due date, and all electronic data will be wiped from the computer hard drive.

**REFERENCES**

Adams HP, Adams RJ, Brott T, Del Zoppo GJ, Furlan A. Guidelines for the early management of patients with ischemic stroke: a scientific statement from the Stroke Council of the American Stroke Association. Journal of Stroke and Cerebrovascular Diseases. 2003; 34: 1056-1083.

Albers GW, Caplan LR, Easton JD, et al. Transient ischemic attack—proposal for a new definition. New England Journal of Medicine. 2002; 347:1713–1716

Centers for Disease Control and Prevention. Prevalence of stroke – United States, 2006-2010. Morbidity and Mortality Weekly Report. 2012; 61: 379-382.

Faulkner J, Lambrick D, Woolley B, Stoner L, Wong L, McGonigal G. Effects of early exercise engagement on vascular risk in patients with transient ischaemic attack and non-disabling stroke. Journal of Stroke and Cerebrovascular Diseases. 2013;: e388-98.

Faulkner J, Lambrick D, Woolley B, Stoner L, Wong L, McGonigal G. The long-term effect of exercise on vascular risk factors and aerobic fitness in those with TIA; A randomized controlled trial. Journal of Hypertension. 2014; 32: 2064-70

Giles M, Rothwell P. Risk of stroke early after transient ischemic attack: a systematic review and meta-analysis. Lancet Neurology. 2007; 6: 1063-1072.

Go AS, Mozaffarian D, Roger VL, Benjamin EJ, Berry JD. Heart disease and stroke statistics – 2013 update: a report from the American Heart Association. Circulation. 2013; 127: e6-e245.

Lozano R, Naghavi M, Foreman K. Global and regional mortality from 235 causes of death for 20 age groups in 1990 and 2010: a systematic analysis for the Global Burden of Disease Study 2010. Lancet. 2012; 380: 2095-2128.

Protogerou AD, Papaioannou TG, Blacher J, Papamichael CM, Lekakis JP, Safar ME. Central blood pressures: do we need them in the management of cardiovascular disease? Is it a feasible therapeutic target? Journal of Hypertension. 2007; 25: 265-72

Roman MJ, Devereux RB, Kizer JR, Lee ET, Galloway JM, Ali T, Umans JG, Howard BV. Central pressure more strongly relates to vascular disease and outcome than does brachial pressure: the strong heart study. Hypertension. 2007; 50: 197-203.

Sharman JE, Marwick TH, Gilroy D, Otahal P, Abhayaratna WP, Stowasser M, et al. Randomized trial of guiding hypertension management using central aortic blood pressure compared with best-practice care: principal findings of the BP GUIDE study. Hypertension. 2013; 62: 1138-1145

Stroke Association (2015) Stroke statistics: January 2015 [online]. Available at: http://www.stroke.org.uk/resource-sheet/state-nation-stroke-statistics (accessed 24/02/2015).

Touze E, Varenne O, Chatellier G, Peyrard S, Rothwell P, Mas J. Risk of myocardial infarction and vascular death after transient ischemic attack and ischemic stroke: a systematic review and meta-analysis. Stroke. 2005; 36: 2748–2755.

Woolley B, Stoner L, Lark S, Wong L, Lanford J, Faulkner J. Effect of early exercise engagement on arterial stiffness in patients diagnosed with a transient ischaemic attack. Journal of Human Hypertension. 2014; 29: 87-91.

Young YY, Abdolhosseini P, Brown F, Faulkner J, Lambrick D, Williams MA, Stoner L. Reliability of oscillometric central blood pressure and wave reflection readings: effects of posture and fasting. Journal of Hypertension. 2015; 33: 1588-1593.
